# Elaborating on theory with middle managers’ experience implementing healthcare innovations in practice

**DOI:** 10.1186/s13012-015-0362-6

**Published:** 2016-01-04

**Authors:** Sarah A. Birken, Lisa D. DiMartino, Meredith A. Kirk, Shoou-Yih D. Lee, Mark McClelland, Nancy M. Albert

**Affiliations:** 1 Department of Health Policy and Management, Gillings School of Global Public Health, The University of North Carolina at Chapel Hill, 1103E McGavran-Greenberg, 135 Dauer Drive, Campus Box 7411, Chapel Hill, NC 27599-7411 USA; 2 Office of Nursing Research and Innovation, Cleveland Clinic, 9500 Euclid Ave., Cleveland, OH 44195 USA

**Keywords:** Middle managers, Nurse managers, Job roles, Healthcare, Innovation implementation

## Abstract

**Background:**

The theory of middle managers’ role in implementing healthcare innovations hypothesized that middle managers influence implementation effectiveness by fulfilling the following four roles: diffusing information, synthesizing information, mediating between strategy and day-to-day activities, and selling innovation implementation. The theory also suggested several activities in which middle managers might engage to fulfill the four roles. The extent to which the theory aligns with middle managers’ experience in practice is unclear. We surveyed middle managers (*n* = 63) who attended a nursing innovation summit to (1) assess alignment between the theory and middle managers’ experience in practice and (2) elaborate on the theory with examples from middle managers’ experience overseeing innovation implementation in practice.

**Findings:**

Middle managers rated all of the theory’s hypothesized four roles as “extremely important” but ranked diffusing and synthesizing information as the most important and selling innovation implementation as the least important. They reported engaging in several activities that were consistent with the theory’s hypothesized roles and activities such as diffusing information via meetings and training. They also reported engaging in activities not described in the theory such as appraising employee performance.

**Conclusions:**

Middle managers’ experience aligned well with the theory and expanded definitions of the roles and activities that it hypothesized. Future studies should assess the relationship between hypothesized roles and the effectiveness with which innovations are implemented in practice. If evidence supports the theory, the theory should be leveraged to promote the fulfillment of hypothesized roles among middle managers, doing so may promote innovation implementation.

## Background

Middle managers—employees who are supervised by an organization’s top managers and who supervise frontline employees [1]—have received increased attention in recent healthcare innovation implementation research [2, 3]. This emerging literature suggests that middle managers influence innovation implementation by bridging informational gaps between top managers and frontline employees that might otherwise impede innovation implementation [1–3]. To leverage middle managers’ influence, it is important to understand how they influence innovation implementation.

The theory of middle managers’ role in implementing innovations in healthcare organizations (“the middle manager role theory”) hypothesized that middle managers promote innovation implementation by fulfilling four roles: diffusing information, synthesizing information, mediating between strategy and day-to-day activities, and selling innovation implementation [1]. It also suggested several activities middle managers might engage in to fulfill the four roles. For example, to diffuse information, middle managers might provide frontline employees with information necessary to implement innovations. Table 1 lists the four roles and examples of activities middle managers might engage in to fulfill the roles.

**Table 1:** Roles and activities hypothesized in the middle manager role theory [1]

Hypothesized activities^a^	Examples of activities identified in this study
Hypothesized role 1: diffusing information	
• Relay information regarding innovation implementation to employees (p 6) • Stay attuned to top managers’ and frontline employees’ moods and needs (p 6) • Provide frontline employees with the information necessary to implement innovations (p 6) • Provide top managers with feedback regarding innovation implementation status (p 6) • Field employees’ questions regarding innovation implementation (p 6) • Inform employees of an innovation that is expected to be implemented (p 6) • Disseminate information regarding material support for innovation implementation (p 6) • Disseminate information regarding emotional support for innovation implementation (p 6) • Disseminate rewards for innovation implementation (p 6)	• Websites • Print/electronic materials • Training • Communicating information (e.g., in-person, meetings, email) • Education
Hypothesized role 2: synthesizing information	
• Make general information about innovation implementation relevant to unique organizations and employees (p 6) • Monitor employees’ responses to the information and reinterpret the information in a way that the employee may find more relevant (p 6) • Use daily conversations to help frontline employees understand key information regarding innovation implementation (p 7) • Interpret facts about innovation implementation may convey to employees the relevance of the innovation to the specific roles that they are expected to fulfill (p 7) • Explain to employees the specific ways in which someone in their role would be supported and rewarded for innovation implementation (p 7)	• Workflow integration • Providing examples
Hypothesized role 3: mediating between strategy and day-to-day activities	
• Give employees the tools necessary to implement innovations (p 7) • Translate information into concrete tasks that must be carried out to effectively implement innovations (p 7) • Provide employees with practical feedback on their innovation implementation-related performance (p 7) • Form “strategic communities” that promote the implementation of new technologies (p 7) • Identify specific activities in which employees are expected to engage to promote an organization’s strategy of innovation implementation (p 7)	• Provide data • Funding • Resources • Assuring right equipment/tools • Support • Workflow integration • Reminders/follow-up
Hypothesized role 4: selling innovation implementation	
• Justify innovation implementation (p 7) • Encourage employees to consistently and effectively use innovations (p 7) • Convince employees that innovation implementation is worthy of their attention (p 7) • Set innovation implementation-related norms (p 8) • Maintain a positive attitude regarding innovation implementation (p 8) • Help employees to appreciate the rationale underlying organizational changes (p 8)	• Describing benefits • Generating “buy-in” from employees • Providing rewards • Reinforcement • Communicating information

^a^Activities intended to fulfill hypothesized roles are described on indicated page numbers in the theory to which we compare middle managers’ experience in practice in this study [1]

Although the theory has received some empirical support [2, 3], the extent to which it aligns with middle managers’ experience in practice is unclear. The objectives of this study were to (1) assess alignment between middle managers’ experience and the theory’s hypothesized roles and activities and (2) elaborate on the theory with examples from middle managers’ experience. Resulting evidence may inform initiatives to promote middle managers’ commitment to innovation implementation [3].

## Method

The study capitalized on a live conference of 154 nurse managers who participated in the Cleveland Clinic’s 2014 Nursing Innovation Summit. The Summit defined, described, and promoted clinical practice innovations (e.g., role redesign on a hospital nursing unit) and described strategies for navigating innovation implementation. During the Summit, SB distributed a self-administered questionnaire that contained closed and open-ended questions. Closed-ended items assessed the extent to which participants’ experience aligned with hypothesized roles and activities (Table 1) [1]. Specifically, we asked participants to:

1. Rate the importance of each of the four hypothesized roles when overseeing innovation implementation in a healthcare organization, using a 7-point Likert scale from 1 (not at all important) to 7 (extremely important) and
2. Rank the relative importance of the four hypothesized roles when tasked with innovation implementation

We used descriptive statistics to summarize perceptions of the importance and relative importance (ranking) of the four hypothesized roles.

Open-ended (qualitative text entry) items allowed participants to elaborate on activities they engaged in to fulfill their roles. Specifically, we asked participants to provide:

1. Examples of activities they engaged in to fulfill each of the four hypothesized roles and
2. Descriptions of activities they engaged in to promote innovation implementation that they perceived to be unrelated to the four hypothesized roles

We employed template analysis, combining content analysis with grounded theory, to analyze participants’ examples of activities engaged in to promote innovation implementation. Content analysis involved using a priori definitions of activities hypothesized in the middle manager role theory; grounded theory allowed activities not hypothesized in the theory to emerge from the analysis [4]. Text units that were aligned with one of the theory’s four roles were coded as such. For instance, “providing in-services” was coded as an activity intended to fulfill the role of diffusing information. Text units that were not aligned with one of the theory’s four roles were coded as emergent and therefore potential elaborations on the middle manager role theory.

The questionnaire was distributed to all 154 Summit participants; of these, 63 were eligible to complete the questionnaire because they identified as ever having been a middle manager and had overseen innovation implementation in a healthcare organization. All eligible participants completed the questionnaire (*n* = 63; response rate = 100 %). The institutional review board at the University of North Carolina at Chapel Hill approved the study and waived written consent due to minimal risks to study participants.

## Findings

### Importance and relative importance of hypothesized roles

Participants generally reported that all roles were “extremely important.” However, one third ranked diffusing and synthesizing information as the most important roles. The majority (60 %) ranked selling innovation implementation as the least important role (Table 2).

**Table 2:** Overall and relative importance of hypothesized roles of middle managers in implementing healthcare innovations (*n* = 63)

Importance of middle manager’s role in implementing innovation (mean score; 1 = not important, 7 = very important)	
• Diffusing information	6.83 (SD = .46)
• Synthesizing information	6.71 (SD = .66)
• Mediating between strategy and day-to-day activities	6.71 (SD = .55)
• Selling innovation implementation	6.63 (SD = .68)
Relative importance of middle manager’s role in implementing innovation (%)	
• Diffusing information	Most important: 31
	Least important: 13
• Synthesizing information	Most important: 34
	Least important: 13
• Mediating between strategy and day-to-day activities	Most important: 24
	Least important: 13
• Selling innovation implementation	Most important: 10
	Least important: 38

### Activities to fulfill roles

Table 1 contains examples of activities participants engaged in to fulfill roles. For example, one participant noted that “remaining positive and encouraging feedback” was important to sell innovation implementation (ID: 31).

Table 3 lists five activities that participants perceived to be unrelated to the four hypothesized roles and suggests how they might elaborate on one of the hypothesized roles. For example, one participant continuously assessed employees’ understanding of the innovation and strategies being used to implement it (i.e., measuring employees’ innovation implementation-related performance) to ensure effective implementation; we posit that measuring employees’ innovation implementation-related performance mediated between strategy and day-to-day activities by giving employees feedback that they could use to improve innovation implementation.

**Table 3:** Emergent activities in which middle managers engage to implement healthcare innovations

Activity	Examples of activity (participant ID)
Hypothesized role 1: diffusing information	
• Obtaining information	• “Research[ing] information [about the innovation]” (10) • “[Conducting a] literature search [about the innovation]” (11)
• Communicating information to external stakeholders	• “Communicat[ing] results [of innovation implementation] to community network” (8)
Hypothesized role 2: synthesizing information	
• Adapting innovation to local context	• “Trouble-shooting” (7) • “Frustrations are expected; time-table change” (11) • “Fine-tuning processes” (52)
Hypothesized role 3: mediating between strategy and day-to-day activities	
• Measuring employees’ innovation implementation-related performance	•“Evaluating work-arounds” (7) • “Establish[ing] benchmarks” (11) • “[Creating an] evaluation tool” (29) • “Evaluat[ing] the process” (43) • “Running…daily data for staff to see” (43) • “Auditing” (44) • “Monitoring compliance” (52)
• Engaging in innovation implementation-related activities	• “Refer[ring] patients to complete treatment” (8) • “[Doing] what the [frontline] staff [do]” (53)
Hypothesized role 4: selling innovation implementation	
None	

## Conclusions

In this study, we sought to elaborate on the middle manager role theory and assess its alignment with middle managers’ experience. Our ability to draw conclusions is limited by our convenience sample and space restrictions for qualitative data entry on the questionnaire; however, study participants shared compelling experiences that are likely to be somewhat consistent with our population of interest: middle managers who oversee innovation implementation in healthcare organizations.

The middle manager role theory generally aligned well with the practical experience of study participants. Of note, participants reported that, in practice, selling innovation implementation is the least important of the four roles. This finding helps to distinguish middle managers from champions. Champions are often top managers or physicians who attempt to persuade employees to engage in innovation implementation (e.g., [5]). Whereas champions may give frontline employees the motivation to implement an innovation, middle managers may give frontline employees the means to do so with practical information, tools, and assistance.

Quantitative and qualitative results differed with respect to participants’ perceptions of the importance of synthesizing information and selling innovation implementation. A third of participants ranked synthesizing information as their most important role, but participants offered the fewest examples of synthesizing information. The opposite is true with selling innovation implementation. It is possible that participants interpreted synthesizing information as a means of selling innovation implementation (e.g., interpreting facts about innovation implementation to convey relevance). On the other hand, middle managers may view selling innovation implementation as champions’ role and, therefore, less central to their own. Despite this, middle managers may engage in selling innovation implementation because they feel better equipped to do so than engaging in roles that they deem more important, such as synthesizing information. Future research should assess whether this is the case; if so, implementation efforts may benefit from training middle managers to engage in the roles that they deem important but are ill-equipped to perform and to delegate roles that they view as less central (e.g., asking champions to sell innovation implementation).

**Fig. 1:**
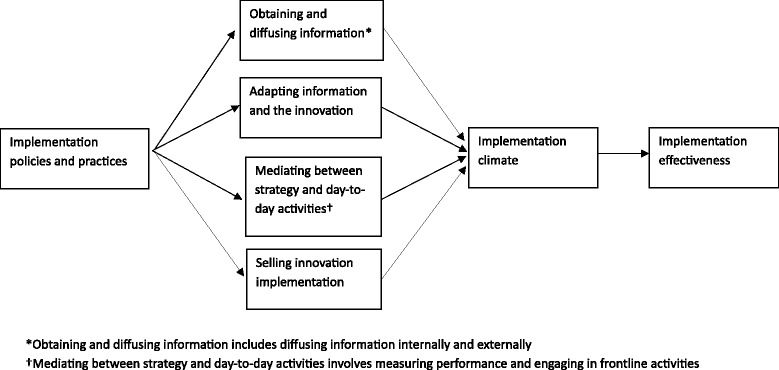
Refined theory of middle managers’ role in implementing innovations in healthcare organizations. *Asterisk* obtaining and diffusing information includes diffusing information internally and externally. *Dagger* mediating between strategy and day-to-day activities involves measuring performance and engaging in frontline activities

Activities participants engaged in that they perceived to be unrelated to the four hypothesized roles expand definitions in the middle manager role theory. Based on our findings, we revised the names and definitions of the four hypothesized roles (Fig. 1). Researchers should assess the relationship between hypothesized roles and implementation effectiveness. If engaging in hypothesized roles promotes implementation effectiveness, then the middle manager role theory should be leveraged to develop an intervention to enable middle managers to fulfill hypothesized roles with the support of top managers [2]; doing so may promote the implementation of innovations intended to reduce the gap between evidence and practice [3].
